# Self-organized intestinal epithelial monolayers in crypt and villus-like domains show effective barrier function

**DOI:** 10.1038/s41598-019-46497-x

**Published:** 2019-07-12

**Authors:** Gizem Altay, Enara Larrañaga, Sébastien Tosi, Francisco M. Barriga, Eduard Batlle, Vanesa Fernández-Majada, Elena Martínez

**Affiliations:** 1 Biomimetic Systems for Cell Engineering Laboratory, Institute for Bioengineering of Catalonia (IBEC), The Barcelona Institute of Science and Technology (BIST), Baldiri Reixac 15-21, 08028 Barcelona, Spain; 2 Advanced Digital Microscopy Core Facility (ADMCF), Institute for Research in Biomedicine (IRB Barcelona), The Barcelona Institute of Science and Technology (BIST), Baldiri Reixac 10-12, Barcelona, 08028 Spain; 3 Institute for Research in Biomedicine (IRB Barcelona), The Barcelona Institute of Science and Technology (BIST), Baldiri Reixac 10-12, Barcelona, 08028 Spain; 4 Centro de Investigación Biomédica en Red de Cáncer (CIBERONC), Barcelona, Spain; 5 ICREA, Passeig Lluís Companys 23, 08010 Barcelona, Spain; 6 Centro de Investigación Biomédica en Red (CIBER), Av. Monforte de Lemos 3-5, Pabellón 11, Planta 0, 28029 Madrid, Spain; 7 Department of Electronics and Biomedical Engineering, University of Barcelona (UB), Martí i Franquès 1, Barcelona, 08028 Spain

**Keywords:** Gastrointestinal models, Intestinal stem cells, Biomedical engineering

## Abstract

Intestinal organoids have emerged as a powerful *in vitro* tool for studying intestinal biology due to their resemblance to *in vivo* tissue at the structural and functional levels. However, their sphere-like geometry prevents access to the apical side of the epithelium, making them unsuitable for standard functional assays designed for flat cell monolayers. Here, we describe a simple method for the formation of epithelial monolayers that recapitulates the *in vivo*-like cell type composition and organization and that is suitable for functional tissue barrier assays. In our approach, epithelial monolayer spreading is driven by the substrate stiffness, while tissue barrier function is achieved by the basolateral delivery of medium enriched with stem cell niche and myofibroblast-derived factors. These monolayers contain major intestinal epithelial cell types organized into proliferating crypt-like domains and differentiated villus-like regions, closely resembling the *in vivo* cell distribution. As a unique characteristic, these epithelial monolayers form functional epithelial barriers with an accessible apical surface and physiologically relevant transepithelial electrical resistance values. Our technology offers an up-to-date and novel culture method for intestinal epithelium, providing an *in vivo*-like cell composition and distribution in a tissue culture format compatible with high-throughput drug absorption or microbe-epithelium interaction studies.

## Introduction

The surface of the small intestine is lined by a monolayer of tightly packed, polarized epithelial cells organized into invaginations called crypts, and finger-like protrusions called villi^1,2^. The intestinal epithelium is a rapidly renewing tissue sustained by the highly proliferative Lgr5^+^ intestinal stem cells (ISCs) that reside at the crypt bases^3,4^. ISCs undergo self-renewal and generate transit amplifying cells, which migrate up the crypt-villus axis and differentiate into absorptive enterocytes, mucus secreting goblet cells, and hormone secreting enteroendocrine cells. While Wingless/Int (Wnt) secreting Paneth cells remain within the ISC niche. After reaching the villi tips, cells undergo apoptosis, are extruded into the lumen, and are replaced by a new cell generation, ensuring intestinal homeostasis^1,2,4^.

*In vitro* research on intestinal epithelium, including studies on basic biology and intestinal disorders, has traditionally been hampered by the lack of appropriated cell culture systems. Conventional models rely on flat two-dimensional (2D) cultures of transformed cell lines such as Caco-2 cells^5,6^. These simplistic models have several shortcomings based on their limited resemblance to normal epithelium. This translates into significant non-physiological values of parameters characterizing their functional properties when compared to the *in vivo* tissue (e.g., underestimated paracellular absorption, abnormally high transepithelial electrical resistance (TEER), and altered expression of metabolizing enzymes)^7,8^. Although physiologically relevant, cultures of primary intestinal epithelial tissues are hardly used *in vitro* due to the swift decrease of proliferative cells and rapid onset of cell death when placed into culture^9,10^.

Recently, technological advances in epithelial cell culture methods have permitted the long-term culture of ISCs with self-renewal and differentiation capacities. It was demonstrated that crypt cells from mouse small intestines organize into three-dimensional (3D) intestinal organoids when embedded in Matrigel, and cultured with biochemical factors mimicking the *in vivo* ISC niche^11,12^. Small intestinal organoids are spherical structures with numerous budding formations. Each of these formations recapitulate the crypt structure, which is composed of dividing cells with Lgr5^+^ ISCs and Paneth cells located at the budding crests. Between budding formations, cells mimic the villus structures, composed of absorptive and secretory cells. The centre of the organoids corresponds to the intestinal lumen, where differentiated cells are spelt upon death. Intestinal organoids can be cultured for several months maintaining highly similar protein expression profiles to freshly isolated crypts^11,12^. Long-term culture of intestinal organoids have been derived from other regions of the mouse intestinal tract^13^ and from other species including humans^14,15^. Undoubtedly, organoids are a breakthrough in cell culture technology, rapidly becoming the gold standard *in vitro* culture method in basic and translational biology studies^16,17^, patient-specific disease modelling^18^, and tissue sourcing for autologous transplantation^19^.

A major drawback of organoids is that their 3D closed geometry impedes direct access to the apical region of the epithelium, which directly contacts dietary factors, external antigens, and microbial components. This limited access prevents organoid routine use in studies of nutrient transportation, drug absorption and delivery, and microbe-epithelium interactions. These applications require technically challenging methods such as organoid-microinjection^20^. Alternatively, methods attempting to open-up the spherical organoids into 2D monolayers allowing for epithelial functional studies have been explored^21–25^. However, these monolayers were not self-renewing, suggesting that stem cells were lost over time. Recent studies report self-renewal properties on epithelial monolayers derived from colonic crypts^26^. The maintenance of the proliferative cell population was attributed to the proper combination of substrate mechanical properties and biochemical factors. These self-renewal characteristics were not reported for small intestine until two very recent studies demonstrated monolayers containing proliferative foci and differentiated zones resembling cell organization *in vivo*^27,28^. Despite attaining a physiologically relevant cell distribution, the reported monolayers neither completely covered the substrate nor allowed access to the cell basolateral side, thus preventing their use in functional tissue barrier assays^27,28^. Such assays are performed in Transwell systems, which provide apical and basolateral compartments separated by cell monolayers grown on porous membranes. Challenges in growing confluent intestinal epithelial monolayers have been attributed to the difficulty in the *in vitro* intestinal cell expansion. Therefore, although there has been progress, an optimal culture method that closely reproduces the *in vivo* intestinal cell composition and distribution while allowing for routine functional tissue barrier assays has not yet been developed.

Here, we describe an experimental protocol that employs mouse-derived small intestinal organoids to obtain intestinal epithelial monolayers that self-organize in crypt and villus-like regions and exhibit effective barrier function. Intestinal cells are grown on substrates coated by thin films of Matrigel, which provide the proper mechanical properties to induce the formation of epithelial 2D monolayers. Live-imaging experiments tracking  green fluorescent protein (GFP)-cells obtained from *Lgr5-EGFP-ires-Cre ERT2* mouse intestines^3^ allow for ISC tracking while epithelial monolayers are growing. These experiments demonstrate that, to grow tissue *de novo*, first ISCs self-organize into crypt-like domains, from which cells migrate out to form non-proliferating and differentiated monolayers, resembling villus-like regions. To expand the culture until confluency, we boost intestinal epithelial proliferation by supplementing the culture medium with intestinal subepithelial myofibroblasts-conditioned medium (ISEMF_CM) and Wnt3a. Upon basolateral administration of this medium, confluent epithelial monolayers with *in vivo*-like cell composition and distribution were reproducibly obtained on Transwells inserts. This enabled analysis of their performance as epithelial barriers with TEER and drug permeability assays. The measured TEER values were within the expected physiological range (40–100 Ω cm^2^) for mouse small intestine^29,30^ and the P_app_ value was comparable to those obtained from human induced pluripotent stem cells (iPSCs)-derived monolayers^31^, indicating an adequate maturation of the epithelium. This technology offers an original, novel tool to generate organoid-derived intestinal epithelial monolayers with *in vivo*-like structural and functional characteristics simultaneously. As such, our experimental set-up can fill in the gap that exists between complex 3D organotypic cell culture systems and 2D formats which are needed for high-throughput testing.

## Results

### Mouse intestinal organoid-derived cells form epithelial monolayers with *in vivo*-like cell type composition and distribution on matrigel-coated hard substrates

Intestinal stem cells (ISCs) maintain their self-renewing and differentiation capacities to form 3D organoids with multicellular organization when embedded in Matrigel drops and cultured with a medium containing specific growth factors^11^. It has been reported that organoids can form 2D epithelial monolayers when cultured on hard substrates coated with Matrigel or collagen^22,27,32^. Recently, both matrix composition and substrate mechanical properties have been identified as key factors in conditioning ISC maintenance and growth^26,28^. We first investigated whether hard substrates would preserve ISC proliferation and differentiation capacities permitting ISC self-organization as *in vivo*. To answer this question, we cultured intestinal organoid-derived crypts or single cells on hard tissue well plates coated with thin films of Matrigel. We decided to employ Matrigel as an extracellular matrix substitute given its molecular composition, similar to the intestinal epithelium basement membrane, and its suitability for ISC growth^11,15,33^. It has been reported that gel thickness less than 20 µm lead to stiffness reaching that of the underlying substrate^34^. We next produced “hard” substrates by coating polystyrene (Young’s modulus of ≈3 GPa)^35^ with thin films of Matrigel 2.9 ± 0.1 µm thick by evenly spreading 10 µL per cm^2^ of substrate (Supplementary Information and Fig. S1). For comparison, “soft” substrates were also produced by coating with thick Matrigel layers (~2 mm in thickness, Storage modulus ≈ 50 Pa)^36^ after depositing 200 µL per cm^2^.

**Figure 1:**
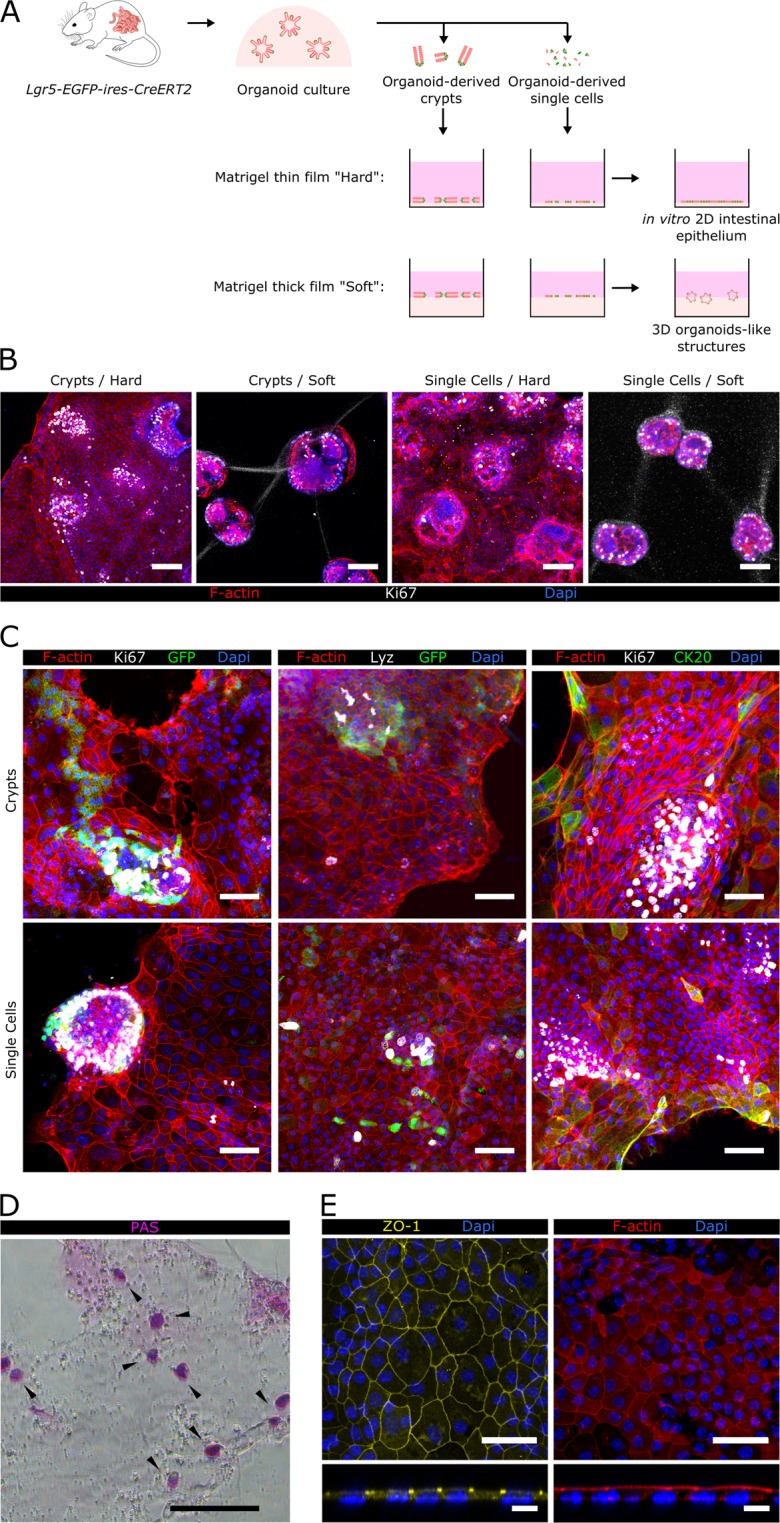
Epithelial monolayers with *in vivo*-like cellular organization are formed on Matrigel-coated hard substrates. (**A**) Scheme depicting the different experimental set-ups employed. Either organoids-derived crypts or single cells where seeded on top of thin or thick layers of Matrigel-coated polystyrene plates. Epithelial structures obtained under each of these conditions are shown. (**B**) Representative images corresponding to co-immunofluorescence of filamentous actin (F-actin) and Ki67 from organoid-derived crypts and single cells cultured either on a thin film of Matrigel, referred as “Hard” substrate, or on a thick layer of Matrigel, referred as “Soft” substrate after 5 days of culture. Scale bars: 100 µm. (**C**) Co-immunofluorescence images for GFP (Lgr5-GFP^+^) and Ki67 (left panel), Lysozyme (Lyz) and GFP (middle panel), and Ki67 and cytokeratin 20 (CK20) (right panel) of epithelial monolayers derived from crypts (upper row) or single cells (lower row) grown on Matrigel-coated hard substrates for 7 days. Scale bars: 50 µm. (**D**) Image of Periodic acid-Schiff base (PAS) staining of epithelial monolayers grown on Matrigel-coated hard substrates for 7 days. Scale bar: 100 µm. (**E**) Immunostaining for *zonula occludens 1* (ZO-1) and F-actin of epithelial monolayers grown on Matrigel-coated hard substrates for 7 days. Upper and lower panels show the top view and the orthogonal cross-sections of the monolayers, respectively. Scale bars: 50 µm (upper panel); 10 µm (lower panel). In all images Dapi was used to stain the nuclei.

Intestinal organoids derived from *Lgr5-EGFP-ires-CreERT2* mice, which express GFP under the Lgr5 promoter, were digested using a mild or harsh digestion protocol to obtain either crypt pieces or single cells, respectively. Both cell fractions were seeded on top of “hard” and “soft” Matrigel-coated substrates (Fig. 1A) and the cell growth was analysed. Actin staining showed that after 5 days of culture both organoid-derived crypt pieces and single cells attached to the “hard” substrates and spread forming an epithelial monolayer. In contrast, neither crypt pieces nor intestinal single cells grew as monolayers on “soft” substrates but formed 3D organoids (Fig. 1B) resembling those obtained in Matrigel drops. These results indicate that substrate stiffness dictates the primary intestinal cell growth phenotype. Immunostaining revealed that intestinal epithelial monolayers formed on “hard” substrates contained proliferative (Ki67 positive) and non-proliferative cells distributed in a clear spatially segregated fashion. Samples were covered by foci of packed proliferating cells separated by non-proliferating areas (Fig. 1B)^28^. Therefore, hard Matrigel-coated substrates provide a suitable environment that favours both ISC division and the formation of an epithelial non-proliferative monolayer.

To characterize the spatially compartmentalized cell composition of the epithelial monolayers, we checked for the expression of intestinal cell-type markers. We found that, regardless of the seeding employed (crypts or single cells), the proliferative areas were formed by a pool of cells double positive for GFP and Ki67, corresponding to actively dividing Lgr5^+^ stem cells (Fig. 1C, left panel). Furthermore, GFP^+^ and Lysozyme^+^ cells are in close proximity (Fig. 1C, middle panel), indicating that Paneth cells form part of the stem cell niche. Since cell composition and distribution of these proliferative areas resemble the *in vivo* intestinal crypts, they are referred to as crypt-like domains. Expression of Cytokeratin 20 (CK20), a marker of mature enterocytes and goblet cells, was found in the non-proliferative areas of the monolayers, showing an increased expression pattern toward the borders of the epithelium, similar to the one found *in vivo*^37^ (Fig. 1C, right panel). Periodic acid-Schiff (PAS) staining showed the presence of mucus secreting Goblet cells scattered throughout the non-proliferative monolayer (Fig. 1D). These features correspond to mature differentiated cells indicting that the non-proliferative areas of the epithelial monolayers correspond to villus-like regions. Accumulation of the tight junction protein *zonula occludens 1* (ZO-1) at the apical inter-cellular membrane, (Fig. 1E, left panel), and of the filamentous actin protein (F-actin) at the cell apical side (Fig. 1E, right panel) demonstrate that in the new-formed epithelial monolayer cells are polarized with the luminal surface exposed to the medium and the basal surface facing the Matrigel. In addition, immunofluorescence against cleaved caspase-3 (CC-3) show that cells underwent apoptosis at the borders of the monolayer (Fig. S2A). Time-lapse microscopy experiments support this finding, as illustrated by a representative time lapse-image sequence where a cell undergoes death and sheds near the epithelial border (Fig. S2B and Supplementary Movie 1), resembling the physiological extrusion of apoptotic cells at the villus tips^38^. Overall, the histological characterization demonstrates that hard Matrigel-coated substrates promote the formation of 2D intestinal epithelium monolayers composed of crypt-like domains containing Lgr5^+^ stem and Paneth cells, and villus-like regions containing mature differentiated epithelial cells, resembling the cellular crypt-villus organization of the *in vivo* intestinal epithelium.

### Cell division in the crypt-like domains fuels the formation of self-renewal organoid-derived intestinal epithelial monolayers

**Figure 2:**
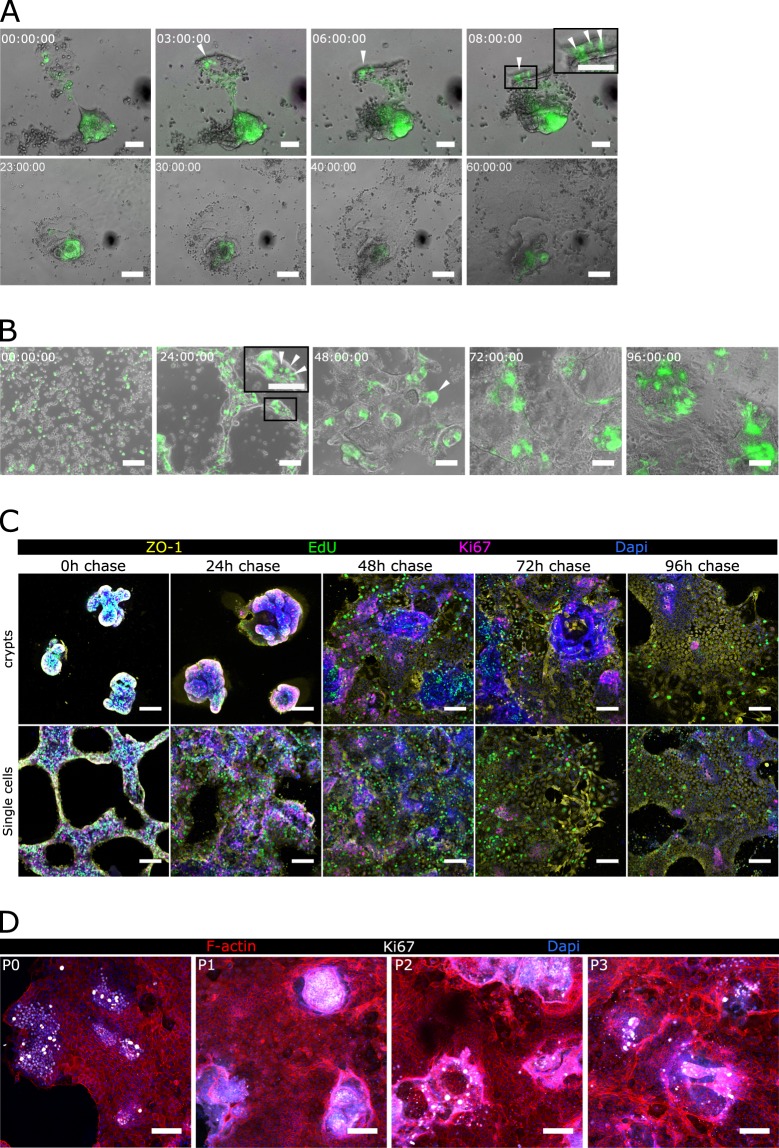
Stem cells self-assemble in crypt-like domains and originate self-sustainable epithelial monolayers. (**A**) Live-imaging sequence of overlapped bright field and GFP signal corresponding to 60 hours after seeding organoid-derived crypts and (**B**) 96 hours after seeding organoid-derived single cells on a thin film of Matrigel. The corresponding time for each snapshot is shown in each panel. White arrow heads indicate Lgr5-GFP^+^ cells. Scale bars: 50 µm (**A**, upper row) and 100 µm (**A**, lower row, and **B**). (**C**) Immunofluorescence of EdU, Ki67, and ZO-1 of an EdU pulse-chase experiment. Representative images of 0 h, 24 h, 48 h, 72 h, and 96 h after EdU chase of either crypt-pieces (upper panels) or single cell (lower panels) derived cultures. Scale bars: 50 µm. (**D**) Immunofluorescence for Ki67 and F-actin of epithelial monolayers from passage 0 to passage 3 (P0, P1, P2, P3). Epithelial monolayers were passed by enzymatical digestion after every 6 to 8 days in culture. Scale bars: 100 µm.

To investigate epithelial monolayer formation on Matrigel-coated hard substrates, we performed ISC cell tracking experiments by monitoring the GFP signal expressed by Lgr5^+^ cells. Time-lapse microscopy experiments show that, after seeding, organoid-derived crypt pieces rapidly refold on themselves to form crypt-like domains containing Lgr5^+^ ISCs, resembling the stem cell disposition at the crypt bases *in vivo* (Fig. 2A, upper row, and Supplementary Movie 2). Right after, cells spread on the substrate and migrated out of crypt-like domains, forming monolayers composed of GFP^−^ cells corresponding to villus-like regions (Fig. 2A, lower row, and Supplementary Movie 2). Notice that the newly formed epithelial layers are not totally flat, but the proliferative regions containing crypt-like structures are slightly elevated with respect to the non-proliferative monolayer (Fig. S3). We then investigated whether the capacity of the intestinal epithelial cells to first self-organize into crypt-like domains and then originate villus-like regions is regulated intrinsically and is not due to the preservation of pre-established crypt configuration. For this, organoid-derived crypts were further digested, disrupting the crypt structure to obtain single cells. We found that upon seeding on Matrigel-coated hard substrates, single cells were able to spontaneously self-assemble to form crypt-like domains containing Lgr5^+^ ISCs (Fig. 2B), closely mimicking the *in vivo* stem cell arrangement at the crypt bases (Fig. 2B, insets). After crypt-like regions were formed, cells started to spread out generating epithelial monolayers composed of GFP^−^ cells, resembling the intestinal villi (Fig. 2B). These results reveal the intrinsic capacity of ISCs to self-assemble and form *in vivo*-like domains when grown on flat, hard substrates. To further characterize the growth of such monolayers, we performed EdU pulse-chase experiments. Organoid-derived crypt pieces and single cells were left to attach on Matrigel-coated hard substrates overnight and then they were pulsed with EdU (0 hours chase). EdU^+^ cells were followed by a chase period of 24, 48, 72 and 96 hours. Immunofluorescence analysis of both EdU and Ki67 showed that, right after the pulse, the forming crypt-like domains were mainly composed of proliferating cells (Ki67^+^) (Fig. 2C, 0 h chase). After one day of chasing, the initially dividing cells started to generate a non-dividing epithelial monolayer (Ki67^−^ EdU^+^) (Fig. 2C, 24 h chase) that continued expanding along the following days (Fig. 2C, 48–96 h chase). The EdU label retention in non-proliferative cells along the culture period analysed demonstrates that cells in the proliferative crypt domains at the EdU pulse time point, give rise to the non-proliferative epithelial monolayer generated during the chase period. Overall, time-lapse microscopy and EdU pulse-chase experiments demonstrate that when grown on Matrigel-coated hard substrates, organoid-derived intestinal cells self-organize into proliferative domains able to generate progeny, which then migrates, differentiates, matures, and dies, closely following the intestinal epithelial homeostasis described *in vivo*.

Furthermore, we demonstrated the maintenance of the proliferative capacities of the generated epithelial monolayers upon sequential passaging. After approximately 1 week in culture, epithelial monolayers were enzymatically digested to obtain single cells that were further cultured on freshly Matrigel-coated hard substrates. These cells were able to regenerate epithelial monolayers maintaining the spatial cell segregation of proliferating foci and non-proliferating regions found in the original monolayer, for up to at least 4 passages (Fig. 2D). The surface coverage of the epithelial monolayers obtained was comparable throughout the passages (~30 mm^2^) (data not shown), indicating the preservation of proliferative capacities of the culture. Therefore, epithelial monolayers generated on Matrigel-coated hard substrates could be passaged and kept in culture for an extended time period without losing their proliferative and self-organizing properties.

### Epithelial monolayers maintain *in vivo*-like cell type composition and distribution on Matrigel-coated Transwell insert membranes

**Figure 3:**
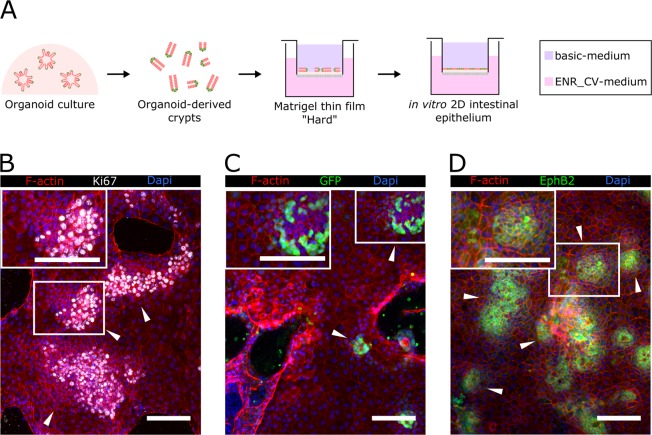
Epithelial monolayers maintain *in vivo*-like cellular organization when grown on Matrigel-coated Transwell insert membranes. (**A**) Scheme depicting the experimental setup. Organoid-derived digested crypts were seeded on top of Matrigel-coated polycarbonate porous membranes and cultured under asymmetric administration of ENR_CV-medium. ENR_CV-medium: EGF, Noggin, R-Spondin 1, CHIR99021 and valproic acid-medium. Representative immunofluorescence images of (**B**) F-actin and Ki67, (**C**) GFP, and (**D**) EPHB2 expressed on epithelial monolayers grown on Matrigel-coated polycarbonate membranes and cultured for 10 days. White arrowheads indicate crypt-like regions. The inset panels in B, C, and D correspond to zoomed images of the crypt-like regions. Scale bars: 100 µm. In all images Dapi was used to stain the nuclei.

Although intestinal organoids are defined as functional mini-organs, in practice assays evaluating their intestinal barrier function are limited. Currently, permeability and absorption assays using intestinal organoids are performed by delivering the tested compound through the epithelium basolateral side^39^. To mimic physiological apical delivery, sophisticated techniques such as intra-organoids microinjection, are needed^40^. Flat monolayers of organoid-derived intestinal epithelium that maintain the cell population and *in vivo*-like distribution while making feasible tissue barrier functional assays are an elegant way to exploit organoid benefits and overcome their steric limitations. Epithelial functional assays are typically based in the standard Transwell insert system, which separates the apical and basolateral compartments of the culture through a monolayer of cells cultured on a porous membrane. Such assays are based on the premise that a mature epithelial monolayer fully covers the membrane forming a selective tissue barrier. We therefore tested whether Transwell hard-porous membranes (polycarbonate polymer, Young’s modulus of ≈4 GPa)^41^ were successful at generating confluent monolayers (Fig. 3A). Organoid-derived crypt pieces were seeded on Matrigel-coated porous membranes and mounted on Transwells. To better mimic the gradients of biochemical factors generated *in vivo* by the stem cell niche^42^, the basolateral compartment was filled in with a medium containing EGF, Noggin, R-Spondin 1, CHIR99021 and valproic acid (ENR_CV-medium) while the apical compartment was filled in with basic-medium (not supplemented with ENR-CV factors) (Fig. 3A). Under these culture conditions, we obtained monolayers that showed spatial cell-type segregation, with proliferative Ki67^+^ crypt-like domains (Fig. 3B), which contained Lgr5^+^ GFP^+^ stem cells (Fig. 3C), and non-proliferative Ki67^−^ villus-like regions (Fig. 3B,C). To further assess the crypt-villus-like cell self-organization of these cultures, we checked for the expression of Eph type-B receptor 2 (EPHB2). EPHB2 receptor is highly expressed in ISCs and is gradually silenced as the cells differentiate^43^. We found high expression of EPHB2 restricted to specific cell clusters distributed throughout the epithelial monolayer (Fig. 3D), similarly to the Ki67 expression, defining the crypt-like domains. EPHB2 expression progressively faded from the centre of these domains (Fig. 3D), suggesting the differentiation of the intestinal cells as they move out of the crypt-like domains, mirroring the *in vivo* cell organization^43^. However, despite physiologically relevant cell composition and distribution, confluent cultures were not achieved. The epithelial monolayers grew rapidly for the initial days of the culture but failed to cover the entire substrate even during extended cell culture periods (up to 4 weeks, data not shown). Similar results were recently reported for small intestinal monolayers^27,28^. Therefore, although successful in recapitulating the main cellular and organizational beneficial features of 3D organoids in 2D systems, the presented methodology does not include epithelial monolayers with tissue barrier properties.

### Biochemical factors produced by intestinal subepithelial myofibroblasts and Wnt3a boost cell growth and result in intestinal epithelial monolayers fully covering transwell insert membranes

**Figure 4:**
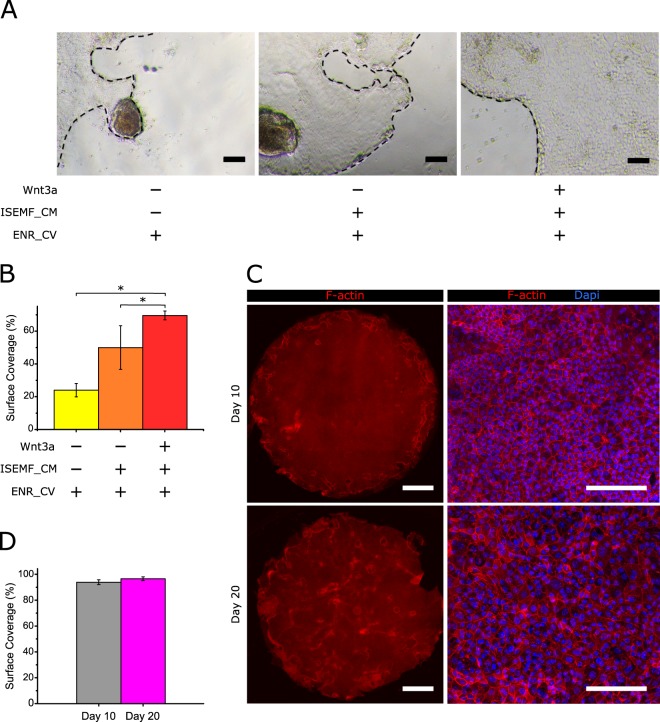
Biochemical factors produced by intestinal subepithelial myofibroblasts and Wnt3a boost epithelial growth. (**A**) Representative bright field images of epithelial monolayers grown on Matrigel-coated polystyrene plates and cultured with ENR_CV-medium, ISEMF_CM supplemented with ENR_CV, and ISEMF_CM supplemented with ENR_CV and Wnt3a after 5 days of culture. Black dashed lines mark the epithelial borders. Scale bars: 100 µm. (**B**) Graph showing the surface coverage percentage (with respect to the total substrate area) for each culture condition. Quantitative data was evaluated from 12 randomly selected regions from bright field images obtained from 3 different samples for each condition (n = 3). The data is represented as mean ± standard deviation. Statistics were performed with Student’s t-test setting *p < 0.05. (**C**) Representative fluorescence images of F-actin and cell nuclei for monolayers cultured in Transwell inserts under the asymmetric administration of ENR_CV-medium supplemented with ISEMF_CM and Wnt3a, at day 10 (upper row) and 20 (lower row) of culture. Left panels show the tile-scan images of the entire Transwell membrane surface (0.33 cm^2^), while right panels show higher magnification images for each condition. Scale bars: 1 mm (left panel) and 100 µm (right panel). (**D**) Graph plotting the surface coverage percentages with respect to the total substrate area as a function of the cell culture time. Six samples were analysed for day 10 (n = 6) and four samples for day 20 (n = 4). ENR_CV-medium: EGF, Noggin, R-Spondin 1, CHIR99021 and valproic acid containing -medium and ISEMF_CM: Intestinal subepithelial myofibroblast-conditioned medium. The data were represented as mean ± standard deviation. Statistics were performed through a Student’s t-test setting *p < 0.05.

To obtain epithelial monolayers fully covering the membranes of Transwell inserts, we focused on increasing the proliferative capacities of the ISCs. One key driver in ISC maintenance and proliferation is the Wingless/Int(Wnt)/β-catenin signalling pathway^1,44^. Paneth cells, which are interdigitated among the ISCs, provide an essential stem cell niche by the constant secretion of Wnt proteins, mainly Wnt3a^45,46^. However, *in vivo* depletion of Paneth cells did not impair the normal intestine homeostasis^47,48^, indicating the redundancy in the Wnt production within the intestinal crypts. Intestinal subepithelial myofibroblasts (ISEMFs) are considered an important alternative non-epithelial source of Wnt pathway activators, including Wnt2, Wnt2b, Wnt4, Wnt-5a, Wnt9b, and Wnt11^46,49,50^, and the enhancers of the Wnt pathway, R-spondin 2 and R-spondin 3^50–52^. Therefore, we postulated that ISEMF-derived signals may be a good strategy to enhance the proliferative capacities of the ISCs in our culture method. To test this hypothesis, ISEMF were isolated from mouse intestines and immunofluorescence staining corroborated the expression of specific markers characterizing myofibroblasts. Cells stained positive for α smooth muscle actin (α-SMA) and vimentin, and negative for desmin, confirmed the ISEMF phenotype^51,53^ (Fig. S4). These freshly isolated ISEMFs were cultured to formulate ISEMF-conditioned medium (ISEMF_CM). We compared the growth of epithelial monolayers on Matrigel-coated hard substrates cultured with the following conditions: ENR_CV-medium, ENR_CV supplemented with ISEMF_CM, and ENR_CV supplemented with ISEMF_CM and recombinant Wnt3a. This last condition was included to further enhance the endogenous Wnt3a signal derived from the Paneth cells of the culture. The effects of the different cell culture media were qualitatively assessed by bright field microscopy (Fig. 4A) and quantitatively evaluated by computing the surface coverage of the monolayers (Fig. 4B). Our results show that, after 5 days in culture, ISEMF_CM increases the surface coverage of the monolayers with respect to the ENR_CV-medium by two-fold. Moreover, adding Wnt3a to this medium further boosts the intestinal epithelium growth by three-fold in surface coverage with respect to regular crypt medium. Considering these results, the culture medium composition was revised to include both ISEMF_CM and Wnt3a in addition to ENR_CV, here after referred as boosting medium.

To test the effect of this approach, organoid-derived crypt pieces were seeded on Matrigel-coated porous membranes and were cultured in Transwells with the new boosting medium added to the basolateral compartment as in previous experiments. Representative images of F-actin immunostaining demonstrate the formation of a tightly packed epithelial monolayer virtually covering 100% of the Transwell membrane area (6.5 mm in diameter) (Fig. 4C, upper panel, and Fig. 4D) at day 10 of culture. We further evaluated the integrity and morphology of these monolayers for longer periods of time, as this is relevant for other potential applications. We find that after 20 days in culture the integrity of the monolayers is preserved (Fig. 4C, bottom left panel) and surface coverage is still complete (Fig. 4D). Thus, our results indicate that the addition of exogenous stem cell niche-derived biochemical factors, including non-epithelial sources, to the regular crypt medium boosts cell proliferation and allows the growth of intestinal epithelial monolayers fully covering the substrate.

### Epithelial monolayers self-organize in crypt and villus-like domains and show effective barrier function

**Figure 5:**
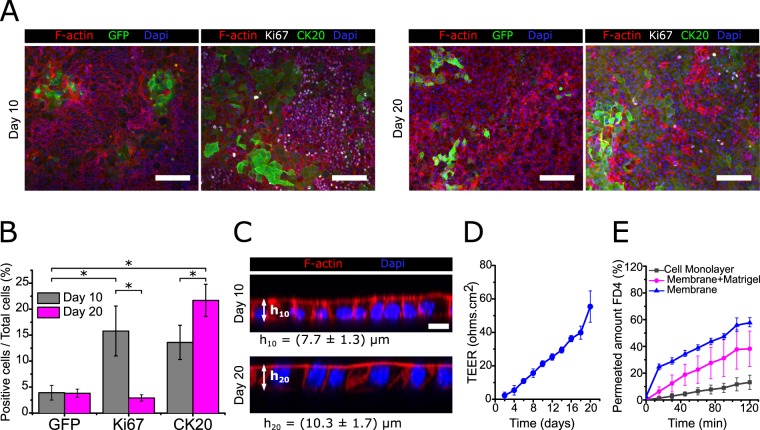
Epithelial monolayers self-organized in crypt and villus-like domains show effective barrier function. (**A**) Representative immunofluorescence images of epithelial monolayers stained for GFP, Ki67, and CK20 after 10 and 20 days in culture on Matrigel-coated Transwell inserts. Scale bars: 100 µm. (**B**) Graph plotting the percentage of positive cells from the total cell number counted for each marker at day 10 (n = 3) and at day 20 (n = 2) of culture. The data were represented as mean ± standard deviation (statistics performed through pairwise Student’s t-test, significance level set at *p < 0.05). (**C**) Representative orthogonal cross-sections of randomly selected flat epithelial monolayers stained for F-actin and nuclei at day 10 and 20 of culture. Scale bars: 10 µm. The average cell heights, represented in the figure, were computed as 7.7 ± 1.3 µm and 10.3 ± 1.7 µm for day 10 (h_10_) and day 20 (h_20_), respectively. For the measurements ten randomly selected cells per sample were measured and a total of three independent samples (n = 3) for each condition were analysed. The data were represented as mean ± standard deviation. (**D**) Plot of the transepithelial electrical resistance (TEER) values measured for the epithelial monolayers as a function of the cell culture time. Reported values are corrected from their corresponding background and account for the total area of the samples. Values are reported as the mean ± standard deviation and correspond to three independent experiments with 5 technical replicas (n = 5). (**E**) Plot of the amount of permeated FITC-dextran 4.4 kDa (FD4) through the epithelial monolayers grown on Transwell inserts. As controls, the flux through the porous Transwell insert membranes coated with a thin layer of Matrigel and through the porous membranes alone was measured. Values are reported as the mean ± standard deviation and correspond to three technical replicas (n = 3).

We analysed the cellular composition and distribution of the intestinal epithelial monolayers generated on Transwell inserts cultured with boosting medium by immunostaining. We found GFP^+^ stem cells clustered in specific areas, corresponding to crypt-like domains containing Lgr5^+^ ISCs, both at day 10 and 20 of culture (Fig. 5A). In contrast, proliferative Ki67^+^ cells were not as clustered as GFP^+^ stem cells, but rather dispersed throughout the monolayer (Fig. 5A). This difference can be attributed to the boosting medium, which increases cell proliferation and may cause the expansion of the transit-amplifying domains out of the GFP^+^ clusters. Differentiated cells marked by CK20 accumulated in non-proliferative areas (Ki67^−^) both at day 10 and 20 (Fig. 5A). Quantification of the different cell populations was performed based on the immunostainings. We find that the amount of GFP^+^ cells remains constant throughout the culture time analysed, indicating that the pool of Lgr5^+^ stem cells is maintained over time (Fig. 5B). Whereas, the relative percentage of proliferative Ki67^+^ cells in the monolayer evolves over time. At day 10, from the percentage of proliferative cells (15.8 ± 4.8%) only about 25% were Lgr5^+^ ISCs (3.9 ± 1.4%), suggesting that the rest of the proliferative population was consisting of transit-amplifying cells. However, at day 20, the percentages of GFP^+^ (3.8 ± 0.8%) and Ki67^+^ cells (2.4 ± 1.2%) were not statistically different (Fig. 5B). In addition, we identified a population of GFP^+^ Ki67^−^ cells, suggesting the presence of non-proliferative stem cells (Fig. S5). Interestingly, this decrease of proliferative cells over time correlates with an increase of differentiated CK20^+^ cells (Fig. 5B). In addition, this is accompanied by a significant increase in the cell height (Fig. 5C), which indicates an enhanced polarization of the epithelial cells forming the monolayer. These results suggest that the epithelial monolayers obtained are highly proliferative at the initial forming stages, while after reaching confluency epithelial differentiation increases, and cell proliferation attenuates.

A Transwell set-up allows for measure transepithelial electric resistance (TEER) measurement to evaluate epithelial monolayer growth and tight junction integrity, both indicators of epithelia barrier function^30,54^. It is a sensitive method, as the presence of small gaps in the epithelial monolayer leads to TEER values near background levels (data not shown). Interestingly, the increase of cell differentiation and polarization over time observed in Fig. 5B,C coincides with an increase in the TEER values throughout culture time (Fig. 5D and Table S1). During the initial stages of the culture, when the epithelial monolayer is forming, and proliferation is still high, TEER values are found to be very low. From day 16 to day 20 of culture, correlating with an increase in intestinal differentiation and maturation of the monolayer, TEER values lay within the physiological range, reported to be between 40 and 100 Ω cm^2^ for mouse small intestine^29,30^.

We further performed permeability studies using FITC-dextran 4.4 kDa (FD4) as a tracer to monitor paracellular transport through tight junctions as another indicators of epithelia barrier function. The amount of FD4 permeated through the epithelial monolayers was significantly lower compared to the controls (Fig. 5E). The apparent permeability (P_app_), calculated using steady state flux, was found to be 1.08·10^−5^ ± 4.50·10^−6^ cm.s^−1^. These results, which differ from non-physiological values usually reported for CaCo-2 cell monolayers^6^, correlate well with the lower TEER values measured for our monolayers (Fig. 5D). In fact, they are comparable to those obtained from human induced pluripotent stem cells (iPSCs)-derived monolayers^31^, in which P_app_ was reported to be in the range of 5.10^−6^–1.10^−6^ cm.s^−1^. Still, the iPSCs-derived system reported a higher TEER value (≈ 600 Ω cm^2^), which can be attributed to a fully differentiated intestinal epithelial monolayer. In contrast, our system displays more physiological permeability and TEER, probably related to the fact that a proportion of stem and transit amplifying is always maintained.

As opposed to other studies in which intestinal differentiation was accomplished by the removal of the activators and the addition of inhibitors of the Wnt pathway^21–23,55^, which may compromise stem cell capacities, our experimental set-up allows for the differentiation and maturation of the epithelial monolayer while preserving a basal level of proliferation and a constant pool Lgr5^+^ ISC cells. The exogenous signals provided by the culture medium employed plus the endogenous signals exerted by the *de novo* forming monolayer contributes to the formation of mature *in vivo*-like epithelial monolayers.

## Discussion

The field of intestinal biology is increasingly interested in intestinal epithelial monolayers that recapitulate *in vivo* physiology to overcome limitations of the closed geometry of organoids. In the last few years, protocols to culture intestinal epithelial monolayers employing primary cells^21–24,56^ have been successfully used in studies of pathogen-epithelium interactions^24,56^ and IgA transcytosis^21^, acknowledging the advantages of epithelial monolayers *versus* closed organoids. However, these methods were focused in achieving fully mature monolayers on Transwell inserts, and self-renewal capacities were not demonstrated. As the culture conditions were optimized for cell differentiation, this may be the cause of the decrease in stem and proliferative cell populations^22,23^. In this context, the development of new robust protocols leading to epithelial monolayers with close to *in vivo* cell composition, distribution, and function will upgrade current *in vitro* models and provide more physiologically relevant results. Here, we show a simple method to obtain mouse small intestine-derived epithelial monolayers organized into proliferative crypt-like domains and differentiated villus-like regions, closely resembling the *in vivo* cell distribution, in a tissue culture format compatible with functional assays. Live imaging experiments employing *Lgr5-EGFP-ires-CreERT2 knock-in* mouse-derived small intestinal cells allowed us to track Lgr5^+^ ISCs during epithelial monolayer formation. Interestingly, we find that Lgr5^+^ cells first reorganized in crypt-like domains that resemble *in vivo* stem cell distribution and morphology, from where cells migrate out to form non-proliferative differentiated villus-like regions. These results show that when ISCs are grown on flat substrates with the proper mechanical stiffness, they retain their capabilities to self-assemble, organize, and give rise to proliferative cells which differentiate, mirroring *in vivo* epithelial physiology. Although recent studies reported similar organization in small intestine epithelial monolayers, their propagation was limited due to the difficulty in cell expansion^27,28^. This is a major drawback which hampers their potential applications in functional barrier assays^57^. Boosting cell proliferation with epithelial Wnt signalling increased proliferation but resulted in the monolayers losing their *in vivo*-like organization^27^. Here, we show that supplementing the crypt medium with additional Wnt sources (intestinal subepithelial myofibroblasts-conditioned medium and Wnt3a), boosted the intestinal cell growth at the early stages of the culture and lead to differentiated and functional monolayers in Transwell inserts. These monolayers display effective barrier function, as demonstrated by the physiological relevant TEER and P_app_ values obtained. During culture, confluence positively correlates with time and we find that the monolayer reaches homeostasis by reducing the percentage of proliferative cells and increases epithelial maturation. Remarkably, this happens in the absence of Wnt inhibitors or Notch activators, in contrast with previously described protocols^21–23^. This can be attributed to different phenomena. In an epithelial regenerative model, it has been shown that non-canonical Wnt-5a, present in ISEMF_CM^46,49^, inhibits the proliferation of ISCs through the activation of transforming growth factor -β (TGF-β) signalling, and allows the formation of new crypts to repair the tissue^58^. Confluent cultures lead to cell cycle exit and promotes differentiation via contact inhibition^59^. Additionally, a negative feedback loop inhibiting proliferation is exerted by the bone morphogenetic proteins (BMPs) generated by the differentiated cells^27^. Therefore, we propose that in our culture set-up the combination of non-epithelial signals provided by the ISEMF-CM and endogenous signals provided by the forming monolayer results in maintaining the homeostasis of the *de novo* epithelium. In line with that, we demonstrated that the percentage of ISCs remained constant throughout the culture time analysed, indicating the self-regulation of the stem cell capacities of these cultures.

In summary, we assert that organoid-derived intestinal epithelial monolayers reported here offer an up-to-date and novel culture method that provides both *in vivo*-like cellular organization and functional barrier properties. In addition, it is a robust culture in a format compatible with studies where access to both luminal and basolateral compartments is needed, such as drug absorption, intracellular trafficking, and microbe-epithelium interaction assays.

## Materials and Methods

### Mouse model

All experimental protocols involving mice were approved by the Animal care and Use Committee of Barcelona Science Park (CEEA-PCB) and the Catalan government and performed in accordance to their relevant guidelines and regulations. *Lgr5-EGFP-IRES-creERT2* mice have been previously described^3^. Briefly, *Lgr5-EGFP-IRES-creERT2* mice were generated by homologous recombination in embryonic stem cells targeting the *EGFP-IRES-creERT2* cassette to the ATG codon of the stem cell marker *Lgr5* locus, allowing the visualization of Lgr5^+^ stem cells with a green fluorescent protein (GFP).

### Intestinal crypt isolation and culture

Intestinal crypts from *Lgr5-EGFP-IRES-creERT2* mice were isolated as previously described^60,61^. Briefly, small intestines were flushed with PBS and cut longitudinally. Villi were mechanically removed, and intestinal crypts were isolated by incubating the tissue with PBS containing 2 mM EDTA (SIGMA) for 30 minutes at 4 °C. The digestion content was filtered through a 70 µm pore cell strainer (Biologix Research Co.) to obtain the crypt fraction. Crypts were plated in Matrigel (BD Bioscience) drops and supplemented with basic-medium: advanced DMEM/F12 (Invitrogen) plus 1% Glutamax (GIBCO), 1% HEPES (SIGMA), Normocin (1:500, Invitrogen), 2% B27 (GIBCO), 1% N2 (GIBCO), 1.25 mM N-acetylcysteine (SIGMA), supplemented with recombinant murine EGF (100 ng ml^−1^, GIBCO), recombinant human R-spondin 1 (200 ng ml^−1^, R&D Biosystems), and recombinant murine Noggin (100 ng ml^−1^, Peprotech) to obtain ENR-medium. The medium was changed every 2 to 3 days. The first 4 days of culture the Rho kinase inhibitor Y-27632 (SIGMA). Outgrowing crypts were passaged once a week and organoid stocks were maintained for up to 4 months.

### Preparation and characterization of matrigel-coated substrates

Ibidi µ-Slides (Ibidi GmbH) or Transwell inserts (6.5 mm diameter, 0.4 µm pore size, Corning) were coated with Matrigel to form thick (~2 mm) and thin (<20 µm) films, by spreading throughout the substrate surface 200 µL (for thick layers) or 10 µL (for thin layers) of Matrigel, followed by an incubation at 37 °C for 1 hour. Matrigel thin films were visualized by immunofluorescence using laminin antibodies, being laminin one of the major components of Matrigel^62^. Matrigel layers were fixed with 10% neutralized formalin (SIGMA), blocked with a blocking buffer containing 1% BSA (SIGMA), 3% donkey serum (Millipore), and 0.2% Triton X-100 in PBS for 2 h and incubated with anti-laminin antibodies (1:500, Abcam) overnight at 4 °C, followed by a secondary antibody Alexa Fluor 488 (1:500, Invitrogen) for 1 h. Images were acquired with a Leica TCS SP5 confocal laser scanning microscope (CLSM) equipped with a 20x dry objective (N.A. = 0.7) and the thicknesses of these layers were estimated using ImageJ. For the detailed explanation of the method used to estimate thicknesses, see the Supplementary Information.

### Generation of organoid-derived intestinal epithelial monolayers

Organoid cultures were enriched for stem cells during 4–5 passages by supplementing the ENR-medium with CHIR99021 (3 µM) and valproic acid (1 mM) to formulate the ENR_CV-medium^63^. To obtain homogeneous sized crypt pieces, full-grown organoids were subjected to a mild digestion protocol. Briefly, Matrigel drops containing organoids were disrupted by pipetting with TrypLE Express1X (GIBCO) and transferred to a Falcon tube at 4 °C, where mechanical disruption, using a syringe with a 23 G 1” needle (BD Microlance 3), was applied. Crypt pieces were seeded, on Matrigel coated substrates, at a cell density of 1000–1500 crypts cm^−2^, using an initial volume of 30–50 µl. Crypt pieces were left to attach to the substrates for 3–4 hours before adding more media. To obtain organoid derived single cells, a harsher digestion protocol was applied by incubating disrupted organoids for approximately 5 minutes at 37 °C. After the digestion, single cells or small clusters of cells were obtained. Cells were seeded, on Matrigel coated substrates, at a cell density of 10^5^ cell cm^−2^, using an initial volume of 10–20 µl. Cells were left to attach to the substrate for 1 h before adding more media. Culture times for each experiment are indicated in the corresponding figure legends. Epithelial monolayers were passed by mechanical disruption followed by enzymatic digestion incubation for approximately 5 minutes at 37 °C with TrypLE Express 1X. Cells obtained from the digestion were reseeded on new Matrigel-coated ibidi µ-Slides. The cultures were passed every 6 to 8 days up to 4 times, maintaining the cells in culture for more than one month. Additional passages were not tested.

### Immunostaining

Cells were fixed with 10% neutralized formalin, permeabilized with 0.5% Triton X-100 and blocked with a blocking buffer containing 1% BSA, 3% donkey serum, and 0.2% Triton X-100 in PBS for at least 2 hours. Primary antibodies used were: anti-GFP (1:100, Life Technologies), anti-GFP (1:100, Abcam), anti-Ki67 (1:100, Abcam), anti-Ki67 (1:100, BD Biosciences), anti-lysozyme (1:100, Dako), anti-*zonula occludens 1* (ZO-1) (1:100, Abcam), anti-cytokeratin 20 (1:100, Dako), anti-Cleaved Caspase-3 (1:200, Cell Signaling Technology), anti-EphB2 (1:200, R&D Systems), anti- α-SMA (1:100, SIGMA), anti- desmin (1:100, Abcam) and anti-vimentin (1:100, Developmental Studies Hybridoma Bank). All samples were incubated with the primary antibody overnight at 4 °C followed by 1 h incubation at room temperature (RT) with secondary antibodies, Alexa Fluor 568 and Alexa Fluor 488 donkey anti-goat, and Alexa Fluor 647 (Jackson ImmunoResearch) diluted at 1:500. Nuclei were stained with 4′,6-diamidino-2-phenylindole (DAPI) (1:1000). Alexa Fluor 568 phalloidin was used to stain filamentous actin (F-actin). For detection of mucus, periodic acid-Schiff (PAS) (SIGMA) was used. The fluorescence images were acquired using either a confocal laser scanning microscopy (TCS SP5, Leica) or an epifluorescence microscope (Axio Observer 7, Zeiss). The bright field images were obtained by an inverted fluorescence microscope (Eclipse Ts2, Nikon).

### EdU pulse-chase experiment for evaluating the monolayer formation

5-ethynyl-2′-deoxyuridine (EdU) staining was performed following the manufacturer’s protocol (Click-iT Plus EdU imaging kit, Invitrogen). Briefly, crypts or single cells were cultured on ibidi µ-Slides coated with Matrigel, explained above. After overnight in culture, cells were incubated with EdU (10 µM) for 2 h, washed with PBS, and cultured with ENR_CV-medium. Samples were fixed with 3.7% formaldehyde (Electron Microscopy Sciences) immediately (chase 0 h) or after 24, 48, 72 and 96 h (chase 24 h, 48 h, 72 h and 96 h) after the EdU pulse. After fixation, samples were permeabilized with 0.5% Triton X-100 in PBS for 20 min and EdU was detected using Click-iT Plus EdU Alexa Fluor 488 imaging kit. Subsequently, the samples were washed with 3% BSA in PBS and incubated with primary antibodies anti-Ki67 (1:100) and anti-ZO-1 (1:200) overnight at 4 °C, followed by incubation with the secondary antibodies, Alexa Fluor 647 and Alexa Fluor 568, for 1 h at RT. Nuclei were stained with Hoechst 33342 (10 µg mL^−1^, Click-iT, Invitrogen) for 30 min at RT.

### Image acquisition and analysis

The fluorescence images were acquired at randomly selected locations using a confocal laser scanning microscope (TCS SP5, Leica) with 10x dry (N.A. = 0.40), 20x dry (N.A. = 0.70), and 40x oil (N.A. = 1.25) objectives. The laser excitation and emission light spectral collection were optimized for each fluorophore, especially for the four-color scans, where the emission bands were carefully adjusted to avoid overlapping channels. The pinhole diameter was set to 1 Airy Unit (AU). For images acquired at 10x and 20x a z-step of 1 µm was used, while for images acquired at 40x we used a z-step of 0.5 µm. The side view images of the monolayer were obtained from the orthogonal views of the confocal microscopy stacks acquired at 40x. The cell heights were measured from the orthogonal views of the stacks at 40x magnification at 10 randomly selected locations for each sample (n = 3). The quantification of the intestinal epithelial cell markers GFP (for stem cells), Ki67 (for proliferative cells), and cytokeratin 20 (CK20) (for terminally differentiated enterocytes and goblet cells), with respect to the total cell number was estimated by a custom-made ImageJ macro. The sequence of operations performed in the macro is given in the supplementary information. For each example, at least five randomly selected locations on the same monolayer were quantified.

### Time-lapse microscopy

Formation of the epithelial monolayer was monitored by time-lapse microscopy, conducted at 10x magnification with Axio Observer 7 epifluorescence inverted microscope (Zeiss) with temperature (37 °C), 95% relative humidity, and CO_2_ (5%) regulation. Phase contrast and EGFP channels were used and images were acquired every 5 min up to 96 h of culture. The time-lapse videos and fluorescence images were processed with ImageJ software.

### Transepithelial electrical resistance (TEER) measurements

To measure TEER values of the epithelial monolayers, Transwell inserts (6.5 mm diameter, 0.4 µm pore size) were coated with thin layers of Matrigel as explained above and seeded with crypts. In these experiments, 30–50 µl of crypt suspension leading to crypt seeding densities of 3000–3500 crypts cm^−2^ were used. Intestinal subepithelial myofibroblast-condition medium (ISEMF_CM) supplemented with ENR_CV and Wnt3A (25 ng mL^−1^), referred as boosting medium, was added to the Transwell compartment corresponding to the cell basolateral side, while basic-medium (without ENR_CV factors) was added to the apical side. TEER was measured with an EVOM2 epithelial voltohmmeter with STX3 electrodes (World Precision Instruments) introduced at the apical and basolateral compartments of the inserts. The electrical resistance corresponding to cell epithelial monolayers was obtained by subtracting the intrinsic resistance (values measured on Transwells with only the porous membranes and the Matrigel-coated porous membranes) from the total resistance measured (including the cells) and corrected for surface area (0.33 cm^2^) expressed as ohm cm^2^.

### Statistical analysis

The data were presented in the figures as mean ± standard deviation. The error bars represent the standard deviations of at least two independent experiments with three technical replicas. Statistical comparisons were performed using two-tailed, unequal variance t-test and a p < 0.05 was considered to be significant.

## Supplementary information

Supplementary Information

Supplementary Movie 1

Supplementary Movie 2

## Data Availability

The datasets generated and analysed during the current study are available from the corresponding author on reasonable request.
