# Author Correction: Self-organized intestinal epithelial monolayers in crypt and villus-like domains show effective barrier function

**DOI:** 10.1038/s41598-019-55181-z

**Published:** 2019-12-06

**Authors:** Gizem Altay, Enara Larrañaga, Sébastien Tosi, Francisco M. Barriga, Eduard Batlle, Vanesa Fernández-Majada, Elena Martínez

**Affiliations:** 1 Biomimetic Systems for Cell Engineering Laboratory, Institute for Bioengineering of Catalonia (IBEC), The Barcelona Institute of Science and Technology (BIST), Baldiri Reixac 15-21, 08028 Barcelona, Spain; 2 Advanced Digital Microscopy Core Facility (ADMCF), Institute for Research in Biomedicine (IRB Barcelona), The Barcelona Institute of Science and Technology (BIST), Baldiri Reixac 10-12, Barcelona, 08028 Spain; 3 Institute for Research in Biomedicine (IRB Barcelona), The Barcelona Institute of Science and Technology (BIST), Baldiri Reixac 10-12, Barcelona, 08028 Spain; 4 Centro de Investigación Biomédica en Red de Cáncer (CIBERONC), Barcelona, Spain; 5 ICREA, Passeig Lluís Companys 23, 08010 Barcelona, Spain; 6 Centro de Investigación Biomédica en Red (CIBER), Av. Monforte de Lemos 3-5, Pabellón 11, Planta 0, 28029 Madrid, Spain; 7 Department of Electronics and Biomedical Engineering, University of Barcelona (UB), Martí i Franquès 1, Barcelona, 08028 Spain

Correction to: *Scientific Reports* 10.1038/s41598-019-46497-x, published online 12 July 2019

In the original version of this Article, Eduard Batlle was incorrectly affiliated with ‘Colorectal Cancer Laboratory, Institute for Research in Biomedicine (IRB Barcelona), The Barcelona Institute of Science and Technology (BIST), Baldiri Reixac 10-12, Barcelona, 08028, Spain’. In addition, two affiliations for Eduard Batlle were omitted. The correct affiliations are listed below:

Institute for Research in Biomedicine (IRB Barcelona), The Barcelona Institute of Science and Technology (BIST), Baldiri Reixac 10-12, Barcelona 08028, Spain

Centro de Investigación Biomédica en Red de Cáncer (CIBERONC), Barcelona, Spain

ICREA, Passeig Lluís Companys 23, 08010 Barcelona, Spain

This error has now been corrected in the PDF and HTML versions of the Article.

