# Supplementary material for: Self-organized intestinal epithelial monolayers in crypt and villus-like domains show effective barrier function
Source: Sci Rep. 2019 Jul 12;9:10140. doi: 10.1038/s41598-019-46497-x (PMC6625996; doi:10.1038/s41598-019-46497-x)
Supplement: Supplementary file 1 — Supplementary Information [file 41598_2019_46497_MOESM1_ESM.docx]

**Supplementary Information**

**Self-organized intestinal epithelial monolayers in crypt and villus-like domains show effective barrier function**

Gizem Altay, Enara Larrañaga, Sébastien Tosi, Francisco M. Barriga, Eduard Batlle, Vanesa Fernández-Majada, Elena Martinez.

**Supplementary Figures**


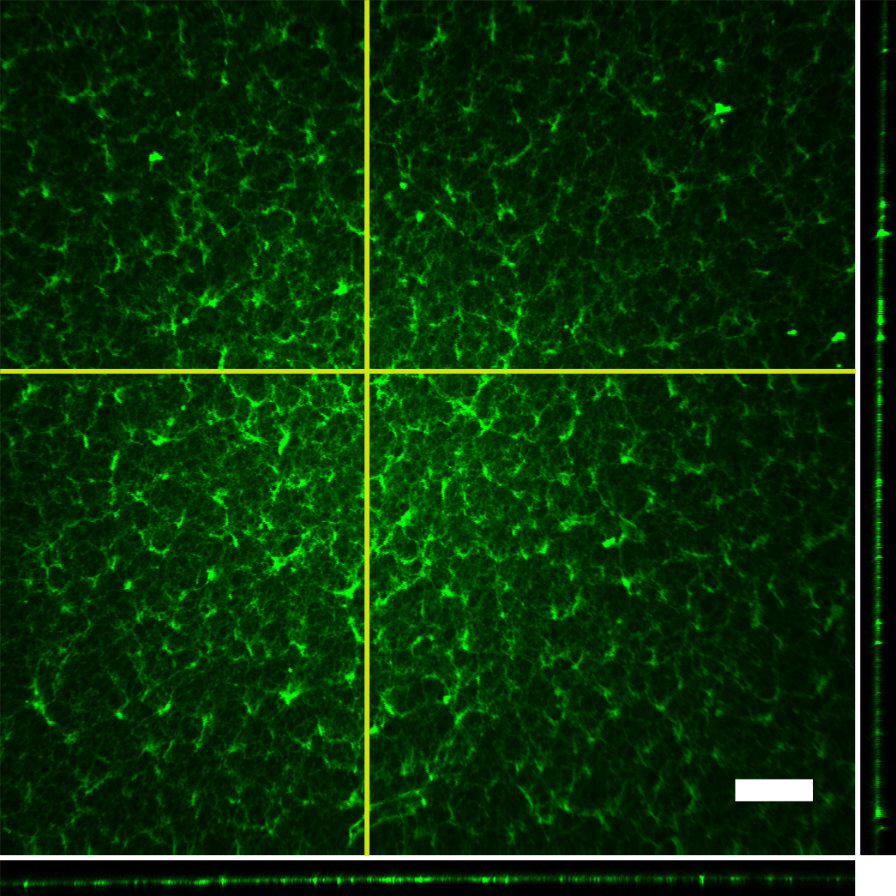


**Figure S1. Characterization of Matrigel-coated substrates.** Immunofluorescence for laminin, one of the more abundant components of Matrigel, of a thin layer of Matrigel on polystyrene plates. One representative top and two orthogonal views are shown. Yellow lines indicate the position from where the orthogonal sections were taken. Orthogonal sections were used to calculate the thin film thickness. Thin film thickness value (2.9 ± 0.1 µm) corresponds to the mean ± standard deviation of measurements obtained from 20 different regions per each sample in a total of three technical replicas (n=3). Scale bar: 50 µm.

**
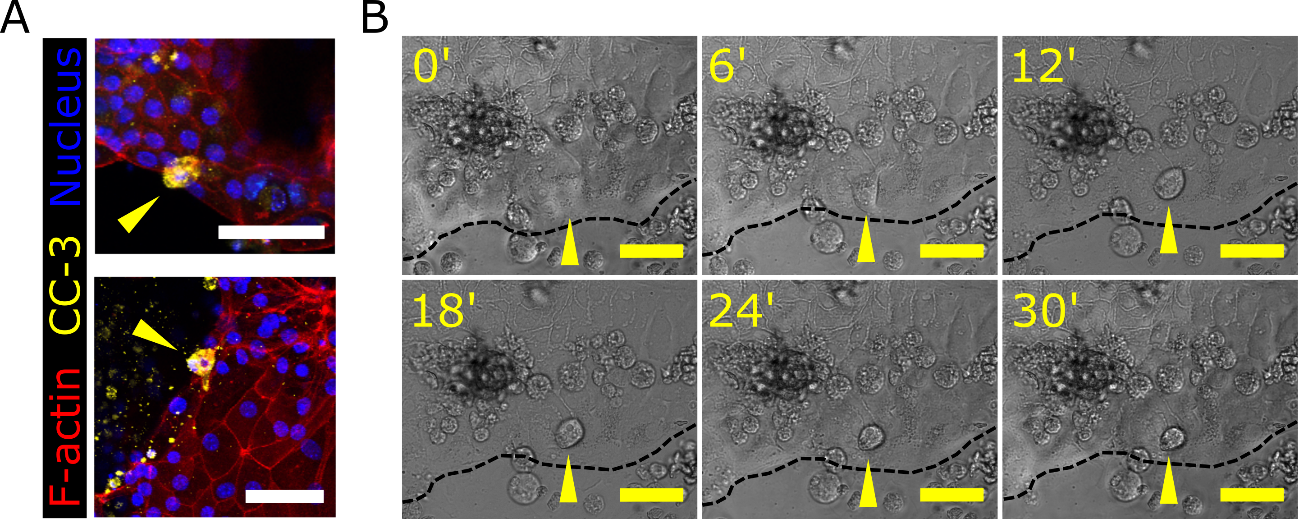
**

**Figure S2. Characterization of cell death** (A) Immunostaining for Cleaved caspase-3 (CC-3) of epithelial monolayers after 7 days in culture showing cell death at the epithelial border. Yellow arrowheads mark apoptotic cells. Scale bars: 50 µm. (B) Live image sequence at day 8 of culture showing a cell dying at the epithelial border. Yellow arrowheads point to the dying cell. Black dashed line marks the epithelial border. Scale bars: 50 µm.


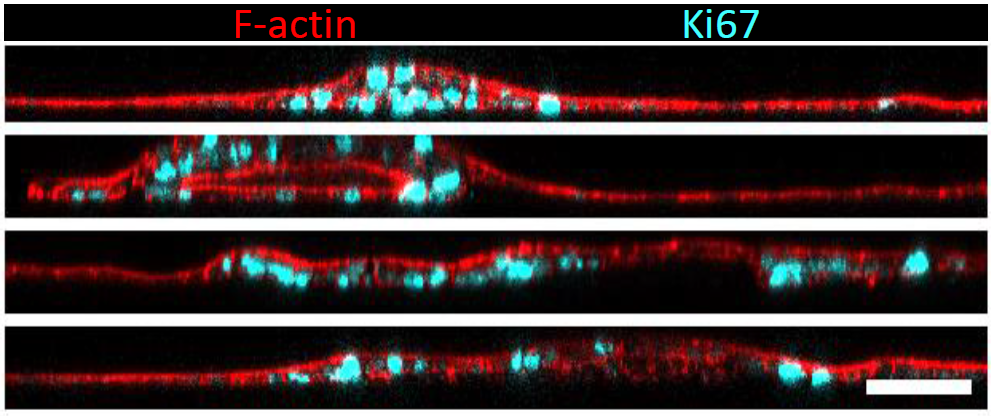


**Figure S3. Topography of epithelial cultures.**

Confocal microscopy cross-sections of immunofluorescence for Ki67 and F-actin showing the bumpy topography coinciding with proliferative regions of the epithelial cultures. Scale bar: 50 µm.


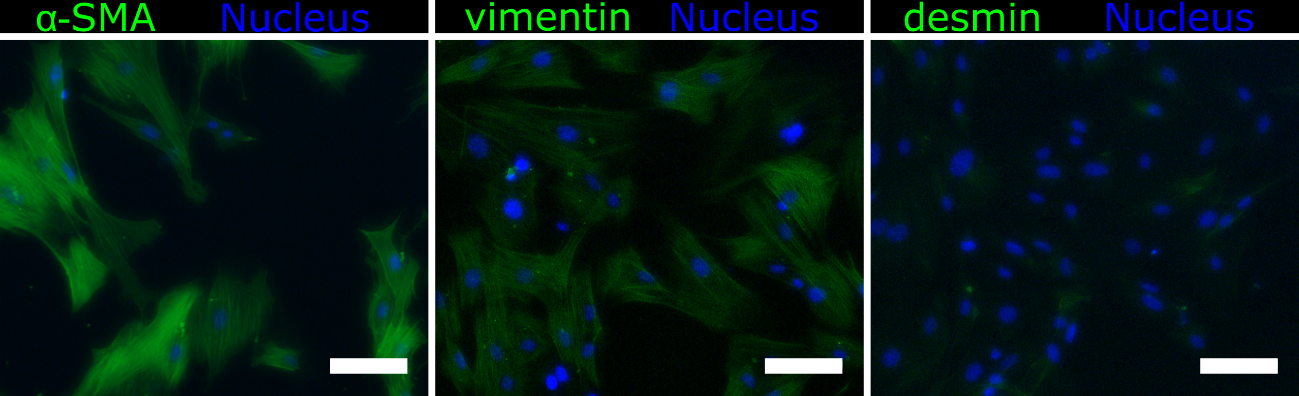


**Figure S4. Characterization of intestinal subepithelial myofibroblasts**

Immunofluorescence for α-smooth muscle actin (α-SMA), and vimentin, and desmin of intestinal subepithelial myofibroblasts (ISEMFs) isolated from mouse colon lamina propria. Counterstaining was done with Dapi. Scale bars: 50 µm.


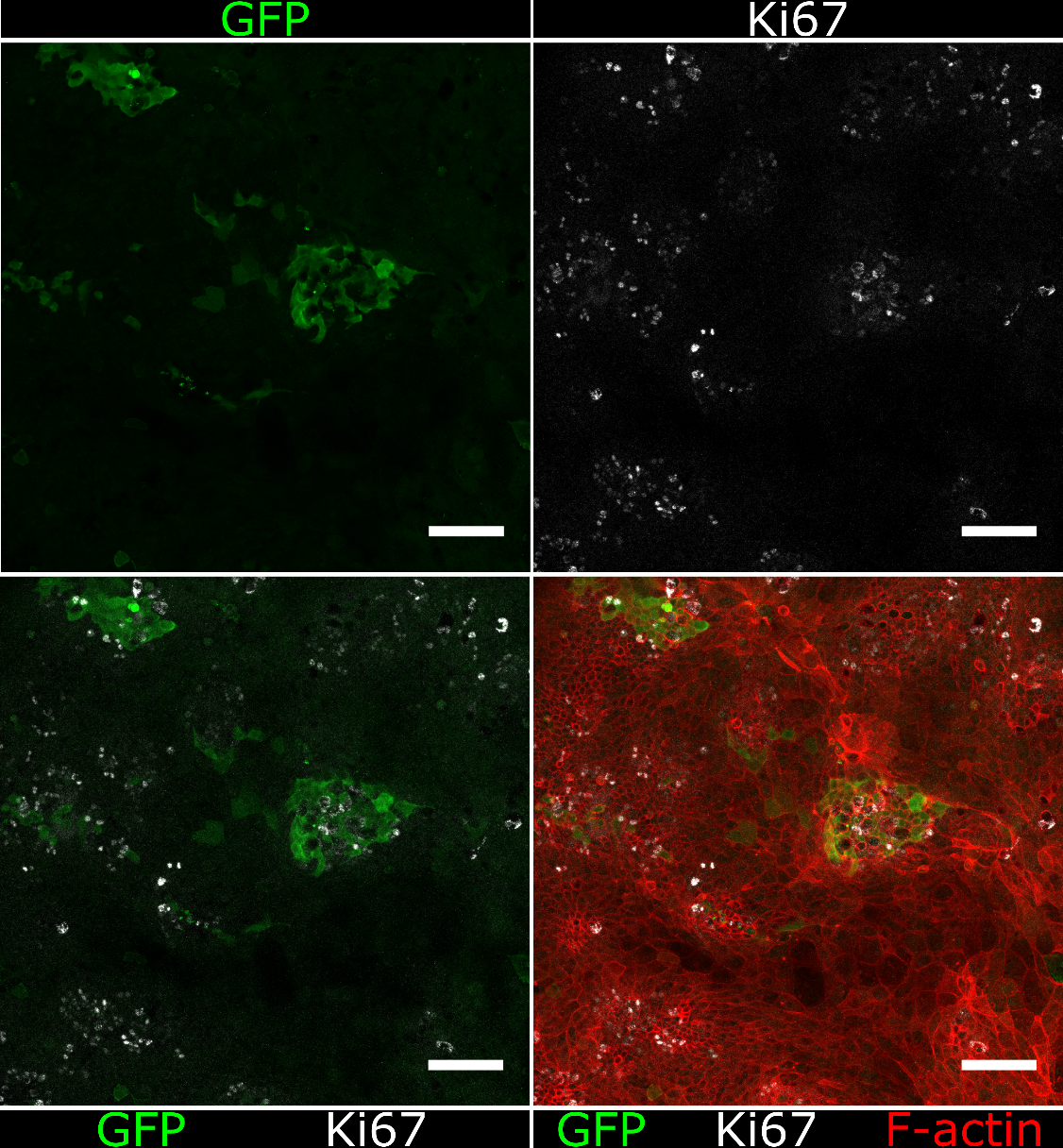


**Figure S5. Characterization of colocalization of GFP^+^ and Ki67^+^ cells in the monolayer**

Representative immunofluorescence images of epithelial monolayers stained for GFP and Ki67 after 20 days in culture on Matrigel coated Transwell inserts. Scale bars: 100 µm.

| Time | Background | | Total Resistance | |
| --- | --- | --- | --- | --- |
| (days) | (ohms.cm^2^) | | | |
|  | mean | sd | mean | sd |
| 2 | 56 | 4 | 58 | 2 |
| 4 | 53 | 3 | 58 | 1 |
| 6 | 50 | 4 | 59 | 6 |
| 8 | 47 | 3 | 62 | 2 |
| 10 | 45 | 3 | 65 | 4 |
| 12 | 43 | 3 | 67 | 3 |
| 14 | 40 | 3 | 68 | 3 |
| 16 | 39 | 3 | 75 | 2 |
| 18 | 38 | 3 | 77 | 5 |
| 20 | 37 | 2 | 94 | 12 |

**Table S1.** Listing the total resistance and background values registered up to 20 days of culture. The background values were subtracted from the total resistance to compute Transepithelial electrical resistance (TEER) of the epithelial monolayers.

**Supplementary methods**

**Intestinal subepithelial myofibroblast isolation and culture**

Intestinal subepithelial myofibroblasts (ISEMFs) were isolated from mouse intestine using a modified version of a previously reported protocol^1^. Briefly, left over tissue from the crypt isolation procedure (above) was further digested by incubation at 37°C for 60 min at 200 rpm with 2000 U of collagenase (SIGMA). The digested tissue was pelleted and lysed at 47ºC for 5 minutes using ACK lysis buffer (GIBCO). The pellet, containing lamina propria cells, was resuspended in Dulbecco’s modified medium (DMEM) (Life Technologies) containing 10% foetal bovine serum (FBS) (GIBCO), 1% penicillin/streptomycin (SIGMA), and 1% minimum essential medium non-essential amino acids (MEM-NEAA) (GIBCO) and cultured in tissue culture plates (25 mm^2^). After 1 week in culture only lamina propria fibroblasts, mainly myofibroblast, remained attached. Plates reached confluence after approximately 20 days in culture. Cell division of primary myofibroblasts are limited, after 6 – 8 passages, cells become senescent. Detection by immunofluorescence of α-smooth muscle actin (α-SMA), vimentin, and reduced desmin expression was used to assess the purity of the myofibroblast cultures. For harvesting the intestinal subepithelial myofibroblast condition medium (ISEMF_CM), plates at 60 – 80% confluence were maintained in culture with the previously described medium for 6 days. Medium was harvested, centrifuged, and filtered using a 22 µm pore strainer, and frozen until use. Medium harvested during multiple days but from the same myofibroblast isolation was pooled to use the same medium in all experiments. ISEMF-CM was complemented with 1% Glutamax, 1% HEPES, Normocin (1:500), 2% B27, 1% N2, 1.25 mM N-acetylcysteine, EGF (100 ng ml^−1^), human R-spondin 1 (200 ng ml^−1^), Noggin (100 ng ml^−1^), CHIR99021 (3 µM), and valproic acid (1mM) before use for organoids cultures.

**Characterization of Matrigel-coated substrates**

Matrigel staining images were acquired by a Leica TCS SP5 confocal laser scanning microscope (CLSM) equipped with a 20x dry objective (NA = 0,7) and hybrid detectors. The laser excitation and emission light spectral collection were both optimized for GFP. The pinhole was set to 1 Airy unit and a stack of 22 µm (z-step of 1 µm) was acquired for each sample. The CLSM stacks were post-processed by deconvolution by a theoretical model of the objective (Mediacy Autoquant X3, fixed PSF, constrained iterative, 10 iterations). This helped mitigating the axial spread of the point spread function of the instrument. We analyzed three technical replicas and the thicknesses were estimated using ImageJ software. To assess the sample uniformity and improve the estimates, for each sample we analyzed 20 randomly selected intensity axial profiles, each extracted along 32-pixel thick lines, covering the whole extension of the stack (ImageJ TransformJ Turn + Plot profile). Since the extracted intensity profiles closely resembled Gaussian functions, we fitted a Gaussian plus offset function to the observations to estimate both the level of the background and the standard deviation of the function (ImageJ Curve Fitting). For each intensity profile, an estimate of the layer thickness was obtained as the full width at half height of the fitted Gaussian function (FWHM ≈ 2.355 σ). The estimates of the layer thickness at different regions were averaged for each sample in a total of three samples and presented as mean ± standard deviation.

**Image analysis**

The quantification of the intestinal epithelial cell markers GFP, Ki67, and CK20, with respect to the total cell number was estimated by a custom-made ImageJ macro. The sequence of operations performed by the macro is as follows. 1) Compute maximum intensity Z-projections (all channels). 2) Apply Laplacian of Gaussian filter to DAPI channel image. 3) Detect salient regional intensity minima in filtered DAPI image. 4) For each fluorophore, measure mean intensity in corresponding channel inside disks centred on detected minima; for disks with mean intensity above user defined thresholds, count nucleus as positive for this fluorophore. Laplacian of Gaussian filter and measurement disk radii were adjusted according to estimated nuclei size at the imaging magnification. Nuclei detection sensitivity (noise tolerance) was adjusted empirically to lead to the most accurate detection. All intensity thresholds were adjusted to local intensity estimates performed inside user selected regions (actin channel, inside tissue; weakest positive cell in label channels). The accuracy of the results was validated from summary images displaying coloured markers at negative/positive nuclei positions overlaid over the projected image stacks. For each sample, at least five randomly selected locations on the same monolayer were quantified.

**Permeability studies**

Permeability studies were done using FITC-Dextran of 4.4 kDa (FD4) (SIGMA) as a tracer to monitor the paracellular transport through the tight junctions. After 21 days in culture, the cells were washed carefully with DMEM without phenol red (GIBCO) supplemented with 1% HEPES and 1% Glutamax (pH 7.4) prior to adding the 200 µL of test compound, FD4, at a concentration of 0,5 mg.mL^-1^ to the apical and 600 µL of DMEM without phenol red (with 1% HEPES and 1% Glutamax) to the basolateral compartments. The cultures were incubated at 37 °C throughout the experiment and samples were taken from the basolateral compartment at specific time points up to 2 h followed by buffer replacement. The fluorescence in the samples was measured using the Infinite M200 PRO Multimode microplate reader (Tecan) at 495 nm excitation and 520 nm emission wavelengths and quantified by a previously established calibration curve with known concentrations. The apparent permeability coefficient (P_app_) was calculated by the following equation:

$$P_{app}=\frac{\mathrm{dQ}}{\mathrm{dt}}.\frac{1}{A.C_{0}}$$

where dQ/dt is the flux, A the area of the filter insert, and C_0_ the initial donor concentration of the test compound. The experiments were performed in triplicate. As controls, the flux through the porous membranes coated with a thin layer of Matrigel and through the porous membranes alone was measured.

1. Khalil, H., Nie, W., Edwards, R. A. & Yoo, J. Isolation of primary myofibroblasts from mouse and human colon tissue. *J. Vis. Exp.* (2013). doi:10.3791/50611
